# Influenza and Bacterial Pathogen Coinfections in the 20th Century

**DOI:** 10.1155/2011/146376

**Published:** 2011-05-11

**Authors:** Xuan-Yi Wang, Paul E. Kilgore, Kyung Ah Lim, Song-Mei Wang, Jeongseok Lee, Wei Deng, Mei-Qi Mo, Batmunkh Nyambat, Jing-Chen Ma, Michael O. Favorov, John D. Clemens

**Affiliations:** ^1^Center for Public Health and Infectious Disease, Institutes of Biomedical Sciences, Fudan University, 138 Yi Xue Yuan Road, Shanghai 200032, China; ^2^Key Laboratory of Medical Molecular Virology of MoH & MoE, Shanghai Medical College, Fudan University, Shanghai 200032, China; ^3^International Vaccine Institute, Seoul 151-818, Republic of Korea; ^4^Laboratory of Medical Molecular Biology, Shanghai Medical College, Fudan University, Shanghai 200032, China; ^5^Department of Biostatistics, School of Public Health, Fudan University, Shanghai 200032, China

## Abstract

To help understand the potential impact of bacterial coinfection during pandemic influenza periods, we undertook a far-reaching review of the existing literature to gain insights into the interaction of influenza and bacterial pathogens. Reports published between 1950 and 2006 were identified from scientific citation databases using standardized search terms. Study outcomes related to coinfection were subjected to a pooled analysis. Coinfection with influenza and bacterial pathogens occurred more frequently in pandemic compared with seasonal influenza periods. The most common bacterial coinfections with influenza virus were due to *S. pneumoniae*, *H. influenzae*, *Staphylococcus spp*., and *Streptococcus spp*. Of these, *S. pneumoniae* was the most common cause of bacterial coinfection with influenza and accounted for 40.8% and 16.6% of bacterial coinfections during pandemic and seasonal periods, respectively. These results suggest that bacterial pathogens will play a key role in many countries, as the H1N1(A) influenza pandemic moves forward. Given the role of bacterial coinfections during influenza epidemics and pandemics, the conduct of well-designed field evaluations of public health measures to reduce the burden of these common bacterial pathogens and influenza in at-risk populations is warranted.

## 1. Introduction

Worldwide, seasonal influenza causes an estimated one million deaths, and *Streptococcus pneumoniae* is associated with approximately 875,000 deaths among children and ~1.1 million deaths among adults each year [1–3]. Influenza and *S. pneumoniae* account for a large proportion of total respiratory disease morbidity and mortality. In addition, bacterial coinfection due to pathogens such as *S. pneumoniae* is a recognized complication of both upper and lower respiratory tract disease due to influenza [4, 5].

With the continued spread of H1N1 influenza virus and the declaration of a global H1N1 influenza pandemic, the impact of this virus may greatly increase in coming months—particularly in populations where there is limited access to health care. In recent years, as pandemic preparedness activities have advanced throughout the world, the treatment, the management, the and prevention of bacterial coinfections (e.g., *S. pneumoniae*, *Haemophilus influenzae* type b [Hib]) have garnered increasing attention [6]. To help understand the potential impact of vaccination against coinfection during pandemic influenza periods, we undertook a broad review of the existing literature that provides new insights into the interaction of influenza and bacterial pathogens.

## 2. Methods

### 2.1. Searching Strategy

In this study, we sought to examine the available evidence from published studies to describe the frequency of bacterial etiologies responsible for coinfection with influenza virus. Studies of the association between influenza and bacterial coinfections, including the impact of *S. pneumoniae* vaccines, were identified using standardized search algorithms for systematic reviews [7, 8]. Published articles in the English and non-English literature were sought through systematic searching of local and international electronic databases. To facilitate identification of published literature, we accessed PubMed (United States), Chinese Bio-Medicine (CBM, China), OVID (Ovid Technologies, Inc., United States), ISI Web of Knowledge (Thomson Reuters, United States), and Korean Medline (KoreaMed, Korea).

The review included articles published between 1918 and 2006. Due to limitations in currently available electronic databases, articles published before 1950 were identified from publication reference lists obtained from scientific periodicals, books, and other publications. Because of the limited availability of electronic citations for studies related to the first influenza pandemic in the 20th century, we performed a hand search covering all issues of JAMA and the Lancet published in 1918 and 1919. Studies of pandemic influenza were identified based on text or data reported in publications that referred to patients who became ill during any of the influenza pandemics of 1918, 1957, or 1968.

To conduct the literature search, medical subject heading (MeSH) terms (influenza, human, pneumonia, bacteria, pneumococcal infections, superinfection, pneumococcal infections, complications, pandemic, and immunization) and free words (coinfection, polymicrobial, predispose, and bacterial coinfection) were used to identify reports. This list of MeSH terms and free words was evaluated in a pilot study to confirm their ability to identify relevant scientific publications. Combinations of these MeSH terms and free words were then constructed for literature searching. In addition, the search terms and their combinations were translated into standard Korean and Chinese medical terminology prior to searching non-English electronic databases.

### 2.2. Reviewing Strategy

Using the databases identified above, all studies published from 1950 to 2006 in English and non-English languages were tabulated for initial review. Studies were excluded from this paper if they met one of the following criteria: (a) had no extractable data or studies limited to single patients (e.g., case reports), (b) had no dates of collection for data reported. Endnote (version X, Thomson, Inc., Philadelphia, USA) bibliographic software was used to create an electronic library of citations identified in our database searches. PubMed searches were performed using Endnote software, and references from each search were imported to Endnote software databases. Study references that could not be uploaded directly into Endnote software (these included references identified in published paper reference lists or identified through other hand searches) were entered manually into study reference databases. After deleting duplicate records, each study was assigned a unique identification code to enable tracking of reviews and analysis. Each citation was then screened by reviewing the text for all report titles and abstracts. Studies that did not meet the inclusion criteria in this study were excluded from the full-text review. All remaining papers and reports underwent full-text review by two independent study reviewers. From each study, the following information was abstracted: design of study, geographic location of study, study time period (month, year), study duration (months or years), total number of study patients, number of patients with bacterial coinfections, types of bacterial pathogens responsible for coinfection, as well as method of influenza virus and bacterial coinfection diagnosis. Kappa (*κ*) statistics were calculated for the interreviewer agreement during the title/abstract and full-text evaluations using Stata 9.2 (StataCorp LP, College Station, Tex). Reports that yielded conflicting information among study reviewers were discussed with coinvestigators to obtain consensus.

### 2.3. Definitions

To identify a case of suspected influenza virus infection, the study had to report patients as having at least one or more of the following clinical signs or symptoms: rapid onset of chills and high fever, frequent epistaxis, myalgia and arthralgia, prostration, pharyngitis without tonsillitis, rhinorrhea and cough with or without sputum, and with or without evidence of chest radiograph abnormalities. A diagnosis of confirmed influenza virus infection was identified when, in addition to the symptoms mentioned above, there was also evidence on laboratory testing of influenza virus infection from (a) a rapid diagnostic test, (b) enzyme immunoassay, (c) isolation of the virus in tissue-cell culture, (d) direct or indirect immunofluorescent antibody staining, (e) reverse transcriptase-polymerase chain reaction (RT-PCR) analysis, or (f) immunohistochemistry. Influenza pneumonia was defined by evidence of an acute pulmonary infiltrate on the chest radiograph. A bacterial coinfection was defined by a positive laboratory test for any bacterial pathogen in a patient with evidence of either clinical or laboratory-confirmed influenza.

### 2.4. Data Analysis

All studies included in this paper underwent data extraction by trained study personnel and data were entered into an MS Excel (Microsoft, Inc., Redmond, Wash, USA) database. SAS statistical software was used for analysis in this study (SAS Institute Inc., Cary, NC, USA). To take into account variations in study designs, diagnostic methods, study periods, and other study characteristics, we utilized a random-effects model to calculate the point estimates of log-transformed proportions (and rates) with their associated 95% confidence intervals [9–11]. The use of the random-effects model allowed for the inclusion of covariates to reduce heterogeneity and for more specific recommendations to be made from this analysis.

Studies were grouped into the following design categories: (a) descriptive studies (e.g., case series, cross-sectional surveys, or surveillance studies), (b) analytic studies (e.g., cohort or case-control studies), and (c) interventional studies (e.g., clinical therapeutic or vaccine trials). To explore the potential association between influenza and bacteria infections, studies were also stratified by type of bacterial pathogen, pandemic period, and type of sample for bacteria culture. The Kruskal-Wallis test was applied (using 0.05 as the level of significance) to compare the difference of *proportions of coinfection* caused by *S. pneumoniae*, *H. influenzae*, *Streptococcus spp*., and *Staphylococcus spp*. during seasonal influenza and pandemic influenza periods.

This study was reviewed and approved by the International Vaccine Institute Institutional Review Board.

## 3. Results

**Figure 1:**
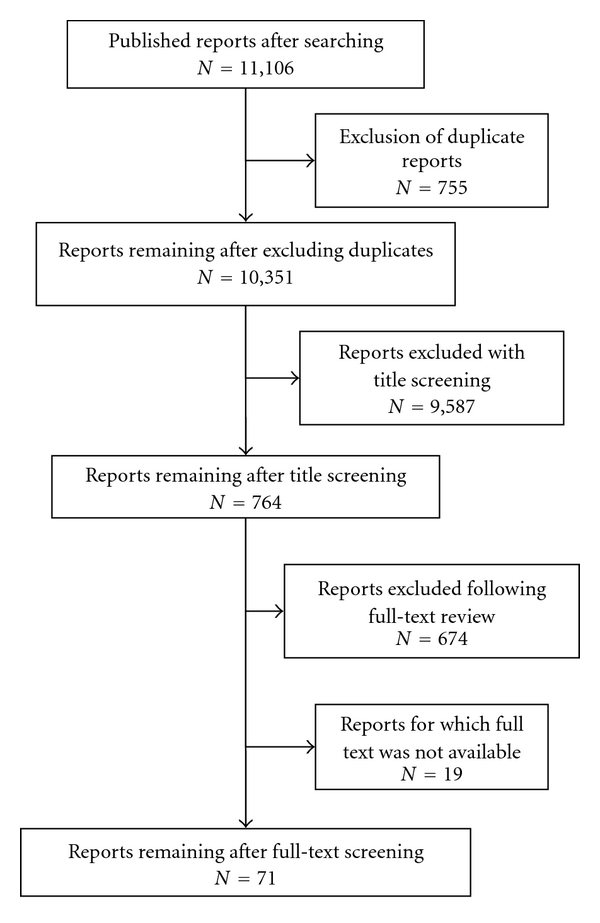
Study flow diagram showing review of reports.

The initial search identified 11,106 influenza and bacteria infection-related citations. After exclusion of duplicate records, a total of 9,587 and 674 studies were excluded using review of title/abstract and full-text information, respectively. Most of studies were excluded due to no extractable data (Figure 1). The interobserver agreement was 86.3% and 92.0% (*P* < .05) for title/abstract and full-text screen, respectively. Seventy-one published studies which met the inclusion and exclusion criteria for this paper were included in the final analysis. Of these reports, 65 studies (91.5%) were descriptive design. Most bacterial infections (93.0%) were diagnosed with culture results, compared to 31.0% cultural diagnosis of influenza (Table 1). Among the 71 articles included in the final analysis, 56 reports presented the data on the association between influenza and bacterial coinfection. Of these, 39 (69.6%) reports originated from either the USA or the UK with the remainder largely from Japan and Spain (Appendix).

A pooled analysis showed that the most common bacterial organisms causing coinfections were *S. pneumoniae*, *H. influenzae*, *Staphylococcus spp*., and *Streptococcus spp*. For all bacterial coinfections, rates of coinfection during pandemic influenza transmission periods were higher than for seasonal influenza (Table 2). *S. pneumoniae* was the most commonly reported (40.8%) bacterial pathogen causing coinfections with influenza during pandemic periods. *H. influenzae* caused coinfection in 12.9% of patients with influenza, while *Staphylococcus spp. *and *Streptococcus spp*. was found in 25.0% and 15.7% of patients, respectively. In addition, studies of seasonal influenza showed that *S. pneumoniae* was the leading cause of bacterial coinfection (pooled average, 16.6%) followed by *Staphylocccus spp*. (6.2%), *H. influenzae* (5.2%), and *Streptococcus spp*. (1.8%).

We conducted further analysis of bacterial coinfections reported during three (1918, 1957, and 1968) influenza pandemic periods (Table 3). Few studies were available from the 1968 pandemic period, and data from these studies did not contain sufficient patient numbers to permit calculation of pooled proportions of coinfection. During the 1918 pandemic, *S. pneumoniae* caused the highest level (56.5%) of coinfection with influenza virus followed by *Streptococcus spp. *(21.7%), *Staphylococcus spp*. (18.8%), and other* H. influenzae* (17.9%). In studies around the 1957 pandemic period, the most common cause of bacterial coinfection with influenza virus was *Staphylococcus spp*. (39.7%) followed by *S. pneumoniae* (15.6%).

In the studies that contained sufficient data for full-text review and data extraction during pandemic period, the proportions of organisms causing coinfections were different while calculated by type of specimens utilized for bacterial diagnosis (Table 4). Studies in which sputum or swab specimens were used for bacterial isolation showed proportions of coinfection ranging from 13.8% for *Staphylococcus spp*., 14.6% for *Streptococcus spp., *14.3% for* H. influenzae,* to 40.8% for *S. pneumoniae* (Kruskal-Wallis test, *P* = .0008). However, among studies that utilized necropsy specimens to detect coinfection, *S. pneumoniae *showed the highest rate (46.5%) of coinfection closely followed by *Staphylococcus spp*. (43.0%), *Streptococcus spp.* (19.6%), and *H. influenzae* (17.6%) (Kruskal-Wallis test, *P* = .02).

## 4. Discussion

The data synthesized in this paper indicate that *S. pneumoniae* is the leading cause of bacterial coinfection during both seasonal and pandemic influenza periods, followed by *Staphylococcus spp*.,* Streptococcus spp*., and* H. influenzae*. However, other major causes of invasive bacterial diseases (e.g., *Staphylococcus spp*.) are close behind *S. pneumoniae* as a cause of coinfection with influenza virus. These data suggest that *S. pneumoniae* and *Staphylococcus spp*. are leading causes of bacterial coinfection with influenza. Interestingly, bacterial coinfection, as well as the relative frequency concluded above, was also demonstrated by several recent studies examining bacterial coinfection during 2009-2010 H1N1 pandemic [12–14]. Notably, the hierarchy of bacterial coinfections identified in this review of studies performed during influenza pandemic periods showed the same order of importance as studies from interpandemic periods. An important observation from our analysis was that the proportion of patients with bacterial coinfection was significantly higher in the pandemic studies compared with studies conducted during interpandemic periods.

Our review has some limitations. First, because of the time period covered by this paper and advancement in laboratory methods over the same period, the laboratory identification of influenza virus and bacterial pathogens varied during the study period. Thus, it is possible that more recent studies had higher sensitivity or specificity for the detection of both viruses and bacteria. In addition, it is possible that other bacterial pathogens may be important but were undetected due to limitations in the laboratory methods used during different time periods or in different countries. In this paper, we found a limited number of cohort studies during either seasonal or pandemic influenza periods. As a result, our analysis could not describe data in a well-defined cohort of influenza patients who were followed prospectively to assess rates of bacterial coinfection. Notwithstanding, the bacterial coinfection and pattern concluded from our analysis were illustrated again during the recent H1N1 pandemic [12–14]. Second, the findings suggest that bacterial coinfection is higher in pandemic periods compared to endemic periods. This observation might be attributable to additional epidemiologic and clinical efforts that are carried out during studies falling within pandemic periods compared with seasonal influenza periods. In fact, out of the 56 studies that provided coinfection data, 34 (60.7%) were conducted during the pandemic period. An additional 22 (39.3%) studies described the pattern of bacterial coinfection during seasonal influenza periods. Moreover, most studies identified in this paper, regardless of whether they were conducted during pandemic or seasonal influenza periods, applied hospital-based designs and focused on severely ill patients with outcomes resulting in hospitalization or death. Finally, the increasing antimicrobial resistance [15, 16] might affect the isolation of bacteria. In this analysis, all pandemic-related studies in this paper were carried out before 1970. Conversely, out of the 22 seasonal influenza studies, 19 (86.4%) were conducted after 1970. Thus, an underestimation of bacterial coinfection in seasonal influenza studies may have occurred where population usage of antibiotics was more prevalent. 

Our analysis suggests that public health measures to reduce the burden of bacterial coinfections is warranted. In a study of pneumococcal vaccine effectiveness in South Africa, it appears that immunization with pneumococcal conjugate vaccine (PCV) in children is associated with moderate protection against influenza [17]. One potential explanation of this effect is that PCV reduces mucosal colonization by pneumococcal vaccine serotypes and engenders herd protection against pneumococcal vaccine serotypes among unvaccinated individuals. In so doing, PCV indirectly reduces severe pneumococcal infections that may be more susceptible to influenza virus infection. A complementary explanation may rest in the fact that PCV directly reduces the burden of severe pneumococcal infections that also reduces the number of individuals in the vaccinated population who are susceptible to influenza virus infection. A limited number of studies have suggested that an excess burden of invasive pneumococcal disease is associated with seasonal influenza epidemics. In Sweden [18], a negative binomial model was used to estimate the excess burden of IPD using influenza and IPD data between 1994 and 2004 from Swedish surveillance system. This analysis showed a yearly increase of 72 to 118 cases of IPD attributable to influenza, which corresponded to 6% to 10% overall per year or 12% to 20% during any given influenza season. Based on our analysis of coinfection studies, *S. pneumoniae* and other bacterial pathogens are likely to reappear as a major cause of bacterial coinfection in future influenza pandemics. Therefore, a key question for policymakers is whether or not vaccines for prevention of invasive bacterial infections caused by *S. pneumoniae* and other pathogens should play a more active role in helping to prepare countries for pandemic influenza. Given the likelihood of continuing influenza virus transmission in present pandemic and bacterial coinfections that occur with influenza, there is an urgent need to reevaluate the full range of tools that may mitigate the burden of invasive bacterial infection, including pneumococcal and Hib vaccines as well as pharmacologic and nonpharmacologic interventions. The evaluation of pneumococcal vaccines with or without influenza vaccine to reduce the burden of coinfections will require large-scale, carefully designed and appropriately powered field trials in order to provide high-quality evidence currently required by public health policymakers.

## Figures and Tables

**Table 1:** Characteristics of studies included in analysis (*n* = 71).

Characteristics	Studies (*n*)	%
*Study type*		
Descriptive	65	91.5
Analytic	2	2.8
Interventional	4	5.6
*Study scope*		
Laboratory-based	1	1.4
Population-based	15	21.1
Hospital-based	55	77.5
*Influenza diagnostic methods*		
Clinical diagnosis	32	45.1
Antigen/antibody detection	16	22.5
RT-PCR	1	1.4
Culture	22	31.0
*Bacterial infection diagnostic methods*		
Clinical diagnosis	3	4.2
Antigen/antibody detection	2	2.8
Culture	66	93.0

Note: RT-PCR, reverse transcription-polymerase chain reaction.

**Table 2:** Pooled results of coinfecting bacterial pathogens identified with influenza virus during seasonal and pandemic influenza periods.

Bacteria	Transmission period	Studies (*n*)	Estimates from random effects model	
			Average % coinfection with influenza virus	95% Confidence interval
*S. pneumoniae*	Seasonal	22	16.6^a^	7.9–31.6
	Pandemic	35	40.8	30.5–52.0
*H. influenzae*	Seasonal	10	5.2^b^	2.3–11.5
	Pandemic	27	12.9	8.3–19.5
*Streptococcus spp.*	Seasonal	7	1.8^c^	0.3–9.3
	Pandemic	27	15.7	9.1–25.8
*Staphylococcus spp*.	Seasonal	12	6.2^d^	2.3–15.7
	Pandemic	26	25.0	15.4–37.8

^a^Seasonal versus pandemic pooled average proportion of patients with *S. pneumoniae* coinfection (*P* = .008, Kruskal-Wallis test; Bonferonni correction, *α* = 0.008).

^b^Seasonal versus pandemic pooled average proportion of patients with *H. influenzae* coinfection (*P* = .02, Kruskal-Wallis test; Bonferonni correction, *α* = 0.008).

^c^Seasonal versus pandemic pooled average proportion of patients with *Streptococcus spp*. coinfection (*P* = .009, Kruskal-Wallis test; Bonferonni correction, *α* = 0.008).

^d^Seasonal versus pandemic pooled average proportion of patients with *Staphylococcus spp*. coinfection (*P* = .005, Kruskal-Wallis test; Bonferonni correction, *α* = 0.008).

^e^During seasonal flu period, the *proportions of coinfection* caused by *S. pneumoniae, H. influenzae, Streptococcus spp*., and *Staphylococcus spp*. were different. (*P* = .009, Kruskal-Wallis test; Bonferonni correction, *α* = 0.008).

^f^During pandemic flu period, the *proportions of coinfection* caused by *S. pneumoniae, H. influenzae, Streptococcus spp*., and *Staphylococcus spp*. were different. (*P* < .0001, Kruskal-Wallis test; Bonferonni correction, *α* = 0.008).

**Table 3:** Comparison of coinfection with major bacterial pathogens and influenza by pandemic period (*n* = 56)^a, b^.

Bacteria	Pandemic period	Studies (*n*)	Estimates from random effects model	
			Average % coinfection with influenza virus	95% Confidence interval
*S. pneumoniae*	1918	23	56.5	45.6–66.8
	1957	9	15.6	8.8–26.0
	1968	3	27.8	2.9–83.2
*H. influenzae*	1918	17	17.9	9.9–30.3
	1957	9	6.9	4.5–10.5
	1968	1	6.3^c^	—
*Streptococcus spp*.	1918	20	21.7	12.9–34.1
	1957	6	4.6	0.6–28.2
	1968	1	9.4^c^	—
*Staphylococcus spp.*	1918	12	18.8	8.0–38.0
	1957	11	39.7	20.9–62.2
	1968	3	10.3	4.9–20.2

^a^Proportions for *S. pneumoniae*, *H. influenzae*, *Streptococcus spp*., and *Staphylococcus spp*. were different (*P* < .0001, Kruskal-Wallis test; Bonferonni correction, *α* = 0.025) during 1918-1919 pandemic.

^b^Proportions for *S. pneumoniae*, *H. influenzae*, *Streptococcus spp*., and *Staphylococcus spp*. were different (*P* = .0006, Kruskal-Wallis test; Bonferonni correction, *α* = 0.025) during 1957 pandemic.

^c^Original data.

**Table 4:** Comparison of coinfection with major bacterial pathogens and influenza by specimen type during pandemic influenza periods^a, b^.

Bacteria	Specimen	Studies (*n*)	Estimates from random effects model	
			Average % coinfection with influenza virus	95% Confidence interval
*S. pneumonia*	Sputum/swab	24	40.8	30.3–52.3
	Sterile fluid	4	7.0	2.1–21.4
	Necropsy	12	46.5	24.8–69.7
*H. influenza*	Sputum/swab	17	14.3	8.6–23.1
	Sterile fluid	2	2.6	0.4–14.6
	Necropsy	9	17.6	6.7–39.1
*Streptococcus spp.*	Sputum/swab	18	14.6	7.1–27.5
	Sterile fluid	2	1.4	0.02–46.4
	Necropsy	12	19.6	9.5–36.2
*Staphylococcus spp.*	Sputum/swab	11	13.8	7.7–23.4
	Sterile fluid	2	2.7	0.5–14.1
	Necropsy	11	43.0	20.8–68.5

^a^Proportions for *S. pneumoniae*, *H. influenzae*, *Streptococcus spp*. and *Staphylococcus spp*. were different (*P* = .008, Kruskal-Wallis test: Bonferonni correction, *α* = 0.025) while utilized sputum/swab specimens to detect coinfection.

^b^Proportions for *S. pneumoniae*, *H. influenzae*, *Streptococcus spp*. and *Staphylococcus spp*. were different (*P* = .02, Kruskal-Wallis test; Bonferonni correction, *α* = 0.025) while utilized necropsy specimens to detect coinfection.
